# Predictors of Sentinel Lymph Node Metastasis in Postoperatively Upgraded Invasive Breast Carcinoma Patients

**DOI:** 10.3390/cancers13164099

**Published:** 2021-08-14

**Authors:** Chi-Chang Yu, Yun-Chung Cheung, Chuen Hsueh, Shin-Cheh Chen

**Affiliations:** 1 Department of General Surgery, Chang Gung Memorial Hospital at Linkou, Chang Gung University, Taoyuan 333, Taiwan; kenneth0609@cgmh.org.tw; 2 Department of Diagnostic Radiology, Chang Gung Memorial Hospital at Linkou, Chang Gung University, Taoyuan 333, Taiwan; alex2143@cgmh.org.tw; 3 Department of Pathology, Chang Gung Memorial Hospital at Linkou, Chang Gung University, Taoyuan 333, Taiwan; ch9211@cgmh.org.tw

**Keywords:** breast cancer, ductal carcinoma in situ, underestimation, sentinel lymph node biopsy

## Abstract

**Simple Summary:**

Routine sentinel lymph node (SLN) biopsy (SLNB) is not necessary for breast-conserving surgery (BCS)-treated ductal carcinoma in situ (DCIS) but is indicated for underestimated invasive carcinoma (IC) postoperatively. In this retrospective study, we aimed to investigate the factors contributing to SLN metastasis in underestimated IC patients with an initial diagnosis of DCIS by core needle biopsy. We found that only the features related to IC, including an IC tumor size (>0.5 cm) and the presence of LVI, could be used as risk predictors of SLN metastasis. In the absence of any predictors, the incidence of positive SLNs was very low (2.6%) in the total population and extremely low (1.3%) in the BCS subgroup. Therefore, we suggest that SLNB may be omitted in patients who initially underwent BCS without risk predictors on final pathological assessment. However, further prospective studies are warranted before its clinical application.

**Abstract:**

Sentinel lymph node (SLN) biopsy (SLNB) usually need not be simultaneously performed with breast-conserving surgery (BCS) for patients diagnosed with ductal carcinoma in situ (DCIS) by preoperative core needle biopsy (CNB), but must be performed once there is invasive carcinoma (IC) found postoperatively. This study aimed to investigate the factors contributing to SLN metastasis in underestimated IC patients with an initial diagnosis of DCIS by CNB. We retrospectively reviewed 1240 consecutive cases of DCIS by image-guided CNB from January 2010 to December 2017 and identified 316 underestimated IC cases with SLNB. Data on clinical characteristics, radiologic features, and final pathological findings were examined. Twenty-three patients (7.3%) had SLN metastasis. Multivariate analysis indicated that an IC tumor size > 0.5 cm (odds ratio: 3.11, *p* = 0.033) and the presence of lymphovascular invasion (odds ratio: 32.85, *p* < 0.0001) were independent risk predictors of SLN metastasis. In the absence of any predictors, the incidence of positive SLNs was very low (2.6%) in the total population and extremely low (1.3%) in the BCS subgroup. Therefore, omitting SLNB may be an acceptable option for patients who initially underwent BCS without risk predictors on final pathological assessment. Further prospective studies are necessary before clinical application.

## 1. Introduction

For patients with a suspicious lesion on breast imaging, the suggested initial diagnostic procedure is imaged-guided percutaneous biopsy rather than surgical biopsy, unless image-guided biopsy is not feasible [1]. Among the various biopsy results, ductal carcinoma in situ (DCIS) accounts for approximately 20% of the newly diagnosed breast cancers [2]. DCIS is a non-invasive breast cancer that occurs within the basement membrane and is defined as no contact with lymphatic vessels. Therefore, pure DCIS is thought to have no theoretical potential for regional lymph node metastasis. Nonetheless, the upgrading rate of DCIS to invasive breast carcinoma (IBC) at final postoperative pathology, initially diagnosed by preoperative percutaneous biopsy, is approximately 23–30% [3]. Associated risk factors for the underestimation of IBC include the biopsy device and guidance method, lesion size on image, tumor grading, mammographic features, and palpable mass [3]. 

For the diagnosis of lymph node stage in patients with early breast cancer and clinically negative axillary lymph nodes (ALNs), traditional ALN dissection [4,5,6] has been replaced by sentinel lymph node biopsy (SLNB) owing to its comparable accuracy, relative safety, and fewer complications. The reported incidence of sentinel lymph node (SLN) metastasis in patients with preoperative DCIS ranges from 0.9–15.6% [7,8,9,10,11,12,13,14]. Although several studies have analyzed the risk factors for subsequent SLN metastasis in patients with preoperative DCIS [7,9,12,14], no other clinicopathological findings or radiological features except for the IBC finding on final pathology, which was consistently confirmed as a factor related to SLN metastasis, were consistently identified by those studies.

According to the American Society of Clinical Oncology guidelines, for patients diagnosed with DCIS preoperatively, SLNB may be performed simultaneously with mastectomy, but it may not be performed for those who will undergo breast-conserving surgery (BCS) [4]. Most studies have discussed the incidence of SLN metastasis in patients diagnosed with DCIS preoperatively, later upgrading to IBC after surgery. These studies included a large number of cases of pure DCIS in the analysis of risk factors for SLN metastasis. Furthermore, the pathological characteristics are usually analyzed using specimens obtained by core needle biopsy (CNB) rather than complete specimens during surgery. However, insufficient tissue obtained by CNB is an important factor for lesions being underestimated as DCIS. In fact, a common clinical scenario is that the lesion is diagnosed as DCIS preoperatively and then diagnosed as IBC after BCS; therefore, SLNB should be subsequently performed following the current guidelines. Since the de-escalation of invasive axillary surgery in clinically node-negative patients with limited SLN metastases is a current trend [15,16]—and three prospective randomized trials, including SOUND, INSEMA, and BOOG13-084 are investigating whether SLNB can be avoided in early breast cancer patients treated with BCS [17,18,19]—it is necessary to consider whether SLNB is unavoidable for all patients with underestimated IBC diagnosed by CNB.

On the basis of these findings, what interested us was whether patients with underestimated IBC diagnosed by imaged-guided CNB must undergo subsequent SLNB examinations regardless of their objective conditions, especially for those who have undergone BCS. The primary objectives of this study were to determine the incidence of SLN metastasis in underestimated IBC patients with DCIS diagnosed by preoperative image-guided CNB and to identify specific clinical characteristics, radiologic features, and final pathological findings that could be predictors of SLN metastasis. A secondary objective was to identify patients who may be treatable without SLNB if the SLN metastasis rate is low. 

## 2. Materials and Methods

### 2.1. Patients 

**Figure 1:**
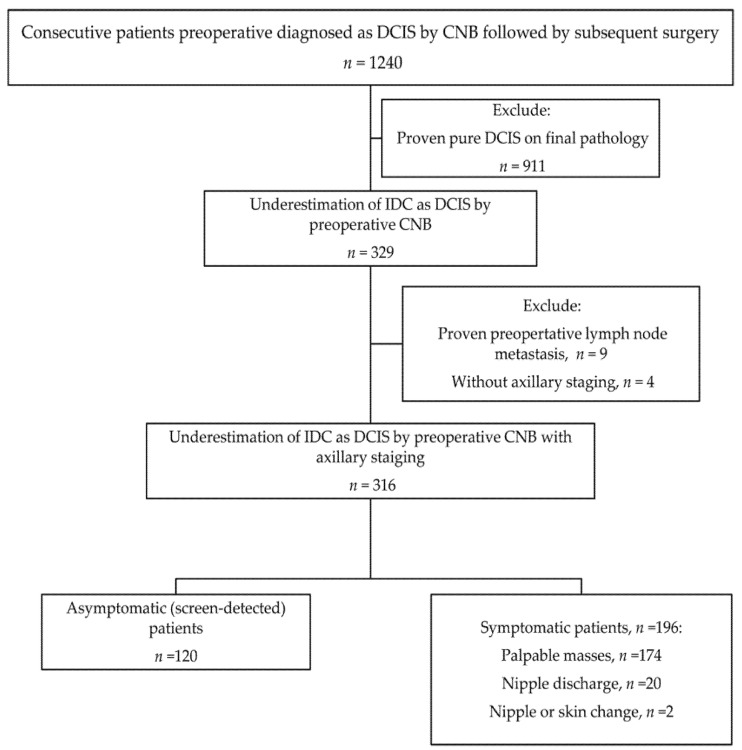
The flow diagram of the study. DCIS, ductal carcinoma in situ; CNB, core needle biopsy; IDC, invasive ductal carcinoma.

We conducted a retrospective review of the electronic medical records in the database of the Department of Pathology at Chang Gung Memorial Hospital, collected between January 2010 and December 2017, and identified 1240 consecutive cases with a preoperative diagnosis of DCIS by image-guided CNB followed by subsequent surgery. The study was approved by the Institutional Review Board of Chang Gung Memorial Hospital, and the need for written informed consent was waived. A single breast cancer pathologist with 28 years of experience reviewed the 1240 CNB results. A total of 329 patients with IBC on the final pathology after subsequent surgery were identified. Nine patients with proven preoperative lymph node metastasis and four with no axillary staging were excluded. The details regarding the data selection are shown in Figure 1. A total of 316 underestimated IBC patients with preoperative DCIS diagnosed by image-guided CNB were finally included in the analysis. Data on clinical findings, patient demographics, radiologic findings, operation type, and pathological findings on final pathology of these cases were collected from the electronic database for analyses.

### 2.2. Image-Guided Core Needle Biopsy Procedure

Ultrasound-guided CNB was routinely performed for suspicious lesions to obtain a tissue diagnosis at our institution. Mammography-guided CNB was preserved for lesions that could only be detected on mammography. Therefore, ultrasound-guided CNB was performed in 275 patients (87.0%), and mammography-guided CNB was performed in 41 patients (13.0%). Ultrasound-guided CNB procedures were performed using a Bard Magnum (Covington, CA, USA) spring-loaded biopsy gun, with a 16-gauge biopsy needle in 267 (97.1%) cases and an 18-gauge biopsy needle in eight (2.9%) cases. Mammography-guided CNB procedures were conducted using mammography with an add-on unit (Lorad, Danbury, CT, USA), with the patient in either a sitting or lateral position, depending on the feasibility of localizing the lesions. Of the 41 mammography-guided CNB procedures, 38 (92.7%) were performed using vacuum-assisted biopsy devices (Vacora or Encor; Bard, Irvine, CA, USA), with a 7-gauge or 10-gauge biopsy needle, and three (7.3%) were performed using a Bard Magnum spring-loaded biopsy gun (Covington, CA, USA) with a 14-gauge biopsy needle. The median number of specimens obtained per lesion was 4 (range, 3–8) under ultrasound guidance and 12 (range, 3–20) under mammography guidance.

### 2.3. Radiologic Features of Tumor

Mammographic images of breast cancer were retrospectively reviewed on a high-resolution digital mammographic screen by a radiologist with 23 years of experience. Ultrasound images of breast cancer were retrospectively reviewed by a sonographer with 15 years of experience diagnosing breast cancer. Both mammography and ultrasound findings were classified according to the 5th edition of the Breast Imaging Reporting and Data System (BI-RADS) [20]. The interpretation of whether the lesion itself can be detected by mammography and/or breast ultrasound was determined by the radiologist and sonographer after discussion. In addition, breast cancer lesions were categorized into three morphologic groups based on mammography and ultrasound manifestations: mass with calcifications, mass without calcifications, and non-mass. 

### 2.4. Surgical Procedure 

Surgical management of breast cancer involves BCS or mastectomy. The choice of operation type is mainly based on the location, size of the tumor, and the patient’s preference. Lymphatic mapping with sentinel lymph node identification was routinely performed using the radiocolloid technique, with subareolar or peritumor injection with technetium 99 m labeled sulfur colloid. 

### 2.5. Pathological Assessment 

SLNs and breast specimens were formalin-fixed, paraffin-embedded, and stained with hematoxylin and eosin (H&E). If no metastasis was detected with H&E staining for SLN analysis, immunohistochemical examination was performed using an anti-cytokeratin antibody. SLNs were classified as negative if they contained no tumor cells and positive if isolated tumor cells (≤0.2 mm), micrometastases (>0.2 mm and ≤2 mm), or macrometastasis (>2 mm) were present. 

The final pathological evaluation of resected breast specimens was reviewed to determine the histologic pattern, DCIS architecture pattern, DCIS and invasive ductal carcinoma (IDC) grade, number of foci of invasion, tumor size of DCIS and IDC, presence of tumor necrosis, presence of lymphovascular invasion (LVI), and immunohistochemistry (IHC) examinations of IBC (estrogen receptor [ER], progesterone receptor [PR], human epidermal growth factor receptor 2 [HER2], and Ki67 index). To identify LVI on H&E sections, we followed the criteria by Rosen PP [21]. First, we tended to focus more on peritumoral areas than intratumoral areas to look for LVI, since shrinkage artifacts resembling lymphatic channels often occurred in the latter. Secondly, true LVI usually did not conform to the shape of the space in which it was found. Thirdly, the space should be lined by endothelial cells to be qualified for a lymphatic channel. IHC stain is of great help in identifying a true lymphatic space and it has been suggested to be a supplementary study in the foregoing guidelines. The DCIS grade was classified as low, intermediate, or high according to the Van Nuys classification [22]. DCIS architecture patterns were classified according to their predominant microscopic growth pattern as comedo DCIS and non-comedo DCIS (including cribriform, solid, papillary, and micropapillary) [23]. Comedo necrosis is conventionally defined as a solid growth of pleomorphic tumor cells with high grade nuclei, central necrosis and sometimes active mitosis. However, there is no consensus regarding the size of the necrosis, which may occupy from 10% to 70% of the ductal diameter [24]. The best cutoff has been proposed to be 53% of the ductal diameter occupied by necrosis [25]. Although the interobserver variability of comedo necrosis is high, we still use the conventional definition to evaluate our cases because no general agreement on size has been established. All IBCs were graded according to the method of Elston and Ellis [26]. Positive ER and PR statuses were defined as tumors with 1% or more positively nuclear-stained cells [27]. HER2 status was considered negative when the IHC score was 0 or 1+. Fluorescence in situ hybridization (FISH) was mandatory in the case of a 2+ IHC score. HER2 positivity was defined as an IHC score of 3 (>10% of cells with strong intensity circumferential membrane staining) or FISH positivity. The cut-off point for Ki67 was 20%.

### 2.6. Statistical Analysis

To assess the association between the variables and the existence of positive SLN in the underestimation of IBC patients diagnosed with DCIS by an ultrasound- or mammography-guided CNB preoperatively, categorical variables were compared using Pearson’s Chi-squared test, and continuous variables were assessed using the t-test. A logistic regression model was used for the multivariate analysis. Differences were considered statistically significant at *p* ≤ 0.05. All statistical analyses were performed using the SPSS software (version 20.0; SPSS Inc., Chicago, IL, USA).

## 3. Results

### 3.1. Clinical Data

The clinicopathologic and radiologic findings are summarized in Table 1. All patients were female, and the median age of the patients was 53 years (range, 27–78 years). Thirty-one patients (9.8%) had a first-degree family history of breast cancer. Of the 316 tumors, 174 (55.1%) were clinically palpable, while the remaining 142 (44.9%) were not.

### 3.2. Radiologic Features

Regarding the imaging modality for detecting lesions, 236 lesions (74.7%) were ultrasonographically and mammographically detectable, 44 (13.9%) were only ultrasonographically detectable, and 36 (11.4%) were only mammographically detectable. The breasts were predominantly categorized into dense (80.6%) and non-dense (19.4%) categories. The imaging morphology of underestimated IBCs was most commonly a mass with calcifications (164/316, 51.9%) and less commonly a mass without calcifications (85/316, 26.9%) or non-mass (67/316, 21.2%). Forty-nine lesions were categorized as BI-RADS 0 on mammography, and their final assessment was performed after ultrasound examination. The main reason for the difficulty in interpreting mammography was the mass (25/49, 51.0%), followed by focal asymmetry (20/49, 40.8%) and architectural distortion (4/49, 8.2%). Regarding the assessment category of the 316 cases based on ultrasound features, 50 (15.8 %) cases were classified as BI-RASDS 4a, 71 (22.5%) cases as 4b, 78 (24.7%) cases as 4c, and 36 (11.4%) cases as 5.

### 3.3. Final Pathological Findings

The median tumor size of DCIS on final pathology was 3.3 cm (range, 0.2–14.0 cm). The DCIS grades were documented to be low in 34 (10.7%), intermediate in 144 (45.6%), and high in 138 (43.7%) patients. The architecture growth pattern of DCIS was composed of 38.6% comedo type and 61.4% non-comedo type. Tumor necrosis was absent in 36 (11.4%) patients and was present in 280 (88.6%) patients. LVI was reported to be absent in most cases (305/316, 96.5%). Of the entire cohort, the histological type of all invasive carcinomas was ductal. Regarding the IDC distribution on the final pathology, a total of 174 (55.1%) cases presented with unifocal lesions, and the remaining 142 (44.9%) cases presented with multifocal or multicentric lesions. A total of about 60% of IDC tumors are less than or equal to 0.5 cm in size. Only four (1.3%) tumors were found, and the size of IDC was larger than the DCIS extent. In terms of IDC tumor grading, 81 (25.6%) cases were defined as grade 1, 133 (42.1%) as grade 2, and 44 (13.9%) as grade 3. ER was positive in 197 (62.3%), PR in 176 (55.7%), and HER-2 in 110 (34.8%) cases. Ki67 was finally evaluated in 291 of these 316 patients, and the remaining 25 cases were non-evaluable because the lesions were too small to be stained. Of the 291 patients, a high Ki67 proliferation index (≥20) was observed in 70 (24.1%) and low (<20) in 221 (75.9%) patients.

### 3.4. Evaluation of Axillary Lymph Node Status

Of the 316 patients, 308 (97.5%) underwent both breast surgery and SLNB simultaneously during the first operation, and the remaining eight (2.5%) underwent SLNB for axillary staging after primary breast surgery. Before 2018, the surgical guidelines for patients with a preoperative diagnosis of DCIS in our institution recommended that a SLNB was indicated in those who underwent mastectomy and should be considered in those with risk factors for an IC component including a palpable mass, high grade DCIS, and large area of mammographic calcifications. A SLNB was also considered in patients who underwent BCS if the tumor location might later interfere with the ability to perform SLNB. The major reason for performing breast surgery and SLNB simultaneously in our population was mastectomy (191/308, 62.0%), followed by a palpable mass (47/308, 15.3%), high grade DCIS (24/49, 7.8%), large area of mammographic calcifications (24/308, 7.8%) and tumor location (22/49, 7.1%). The median number of SLNs removed was 2 (range, 1–8). The SLNs showed macrometastasis in 15 (4.7%), micrometastasis in seven (2.2%), isolated tumor cells in one (0.3%), and no metastasis in 293 (92.7%) patients (Table 2). Among the 23 patients with positive SLNs, 17 underwent completion ALN dissection. In 11 out of 17 patients (64.7%), no additional lymph node metastases were found and, in six, micrometastases were found. Of the six patients, four had only one additional positive lymph node and two had extensive nodal disease, with three and 11 additional positive lymph nodes, respectively. Six patients with positive SLNs did not undergo ALN dissection, four of them had micrometastases, one had macrometastasis, and the remaining one had isolated tumor cells.

### 3.5. Predictors of Sentinel Lymph Node Metastasis

On comparing the clinicopathological findings and radiologic features between patients with negative SLNs and those with positive SLNs, we found significant intergroup differences in IDC tumor size and the presence or absence of LVI. Compared with the patients in the negative SLN group, patients in the positive SLN group were more likely to have an IDC tumor size > 0.5 cm (17/23, 73.9% versus 106/293, 36.2%; *p* < 0.001) and the presence of LVI (8/23, 34.8% versus 3/293, 1.0%; *p* < 0.0001) (Table 3). Among all the clinical variables examined, there were no significant intergroup differences in median patient age, lesion location, and first-degree family history of breast cancer. Although a clinically palpable mass was more common in patients with positive SLNs (17/23, 73.9%) than in those with negative SLNs (157/293, 53.6%), the difference was not statistically significant (*p* = 0.059). Neither radiologic features nor biopsy procedure-associated variables were found to be predictors of SLN metastasis. Mastectomies were performed more frequently in the positive SLN group (78.3%) than in the negative SLN group (59.0%), but the difference was not significant (*p* = 0.070). The two groups were comparable in terms of DCIS tumor grade (*p* = 0.797), DCIS architecture pattern (*p* = 0.084), DCIS tumor size (*p* = 0.117), tumor necrosis area (*p* = 0.912), IDC tumor grade (*p* = 0.165), ER status (*p* = 0.458), PR status (*p* = 0.340), HER2 status (*p* = 0.647), and Ki67 (*p* = 0.873). 

Multivariate analysis demonstrated that an IDC tumor size of >0.5 cm (odds ratio: 3.11; confidence interval: 1.09–8.81; *p* = 0.033) and the presence of LVI (odds ratio: 32.85; confidence interval: 7.56–142.80; *p* < 0.0001) were independent risk predictors of SLN metastasis (Table 4). 

**Figure 2:**
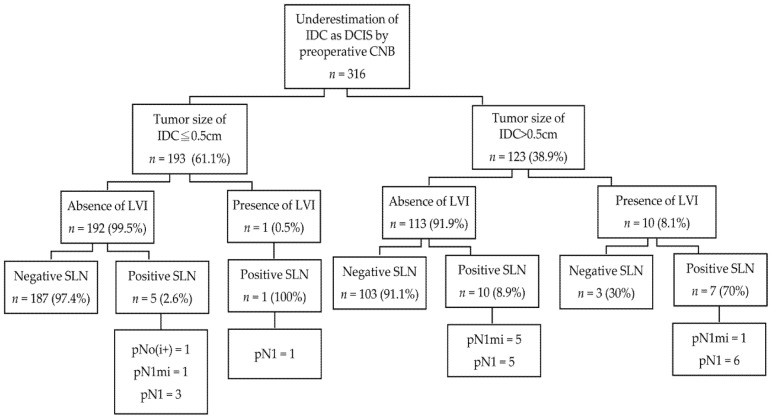
Results of sentinel lymph node biopsy stratified based on tumor size of invasive ductal carcinoma and lymphovascular invasion. IDC, invasive ductal carcinoma; DCIS, ductal carcinoma in situ; CNB, core needle biopsy; LVI, lymphovascular invasion; SLN, sentinel lymph node; pN0(i+), isolated tumor cells; pN1mi, micrometastasis; pN1, 1–3 macrometastasis.

The results of SLNB stratified based on tumor size and LVI are shown in Figure 2. In the current study, the incidence of negative SLNs with tumors that met the features of small IDC tumor size (≤0.5 cm) and absence of LVI was as high as 97.4% (187/192); in contrast, the incidence of tumors that did not meet the above features with negative SLN was only 85.5% (106/124). 

**Figure 3:**
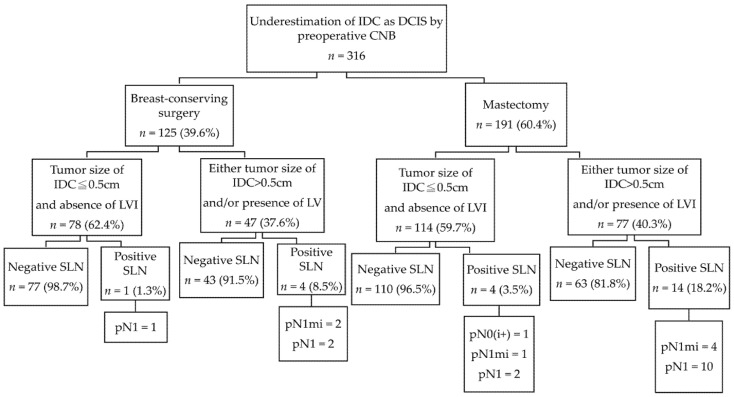
Results of sentinel lymph node biopsy according to different surgical procedures and the combination of tumor size of invasive ductal carcinoma (≦0.5 cm) and absence of lymphovacular invasion. IDC, invasive ductal carcinoma; DCIS, ductal carcinoma in situ; CNB, core needle biopsy; LVI, lymphovascular invasion; SLN, sentinel lymph node; pN0(i+), isolated tumor cells; pN1mi, micrometastasis; pN1, 1–3 macrometastasis.

The results of SLNB according to different surgical procedures and the combination of tumor size of IDC (≤0.5 cm) and absence of LVI are shown in Figure 3. In patients undergoing BCS, an extremely low incidence of positive SLNs of 1.3% (1/78) was observed in subgroups without any predictor.

**Figure 4:**
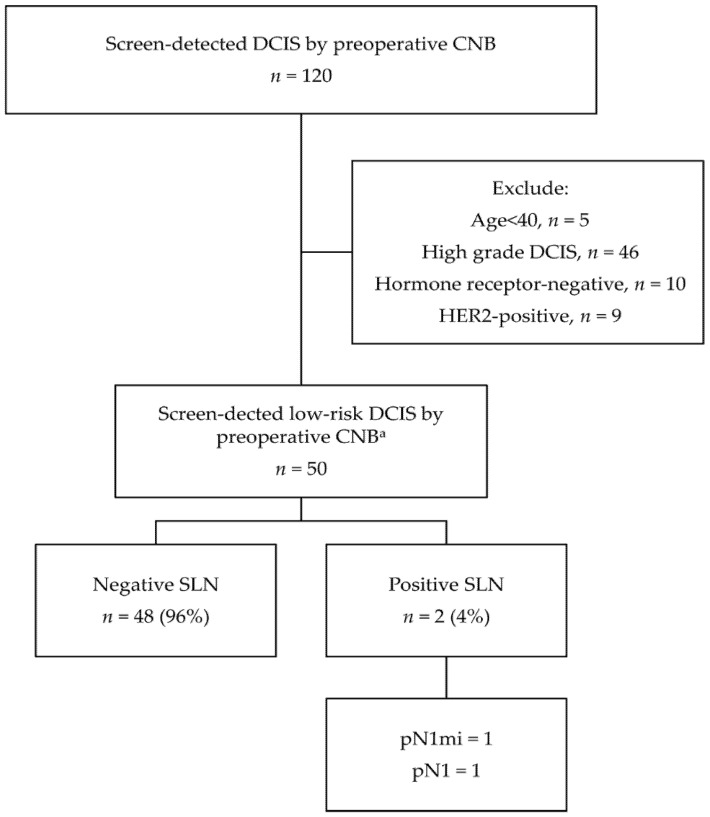
Results of sentinel lymph node biopsy among patients diagnosed with low-risk ductal carcionm in situ preoperatively. DCIS, ductal carcinoma in situ; CNB, core needle biopsy; HER2, human epidermal growth factor receptor 2; SLN, sentinel lymph node; pN1mi, micrometastasis; pN1, 1–3 macrometastasis. ^a^ The definition of low-risk DCIS is consistent with the inclusion criteria of the COMET(Comparison of Operative versus Monitoring and Endocrine Therapy) trial.

An incidence of positive SLNs of 4% (2/50) was observed in patients diagnosed with low-risk DCIS, preoperatively in our series (Figure 4). The criteria for defining low-risk DCIS were the same as in the COMET (Comparison of Operative versus Monitoring and Endocrine Therapy) trial, including 40 years of age or older, low/intermediate DCIS without IBC diagnosed on CNB, ER(+) and/or PR(+), HER2(−) and no mass on physical examination or imaging [28].

To investigate whether different predictors existed for aymptomatic (screen-detected) population, we conducted analyses of factors associated with positive SLNs and found a statistically significant correlation of positive SLNs with an IDC tumor size > 0.5 cm (4/5, 80.0% versus 35/115, 30.4%; *p* = 0.038) and the presence of LVI (1/5, 20.0% versus 0/115, 0%; *p* = 0.042) (Table S1). The confirmed predictors associated with positive SLNs in asymptomatic breast cancer patients were the same as those associated with positive SLNs in total population (including asymptomatic and symptomatic breast cancer patients).

## 4. Discussion

The overall underestimation rate of DCIS patients by preoperative CNB was 26.5% (329/1240) in our cohort, which was comparable to the 25.9% reported in a previous meta-analysis study containing 7350 cases [3]. In the entire study population of 316 patients with underestimated IBC diagnosed by imaged-guided CNB, the incidence of SLN metastasis was 7.3%, which is within the reported incidence ranging from 0.9–15.6% [7,8,9,10,11,12,13,14] in patients with preoperative DCIS. It is noteworthy that, unlike previous study objects, which were involved in many pure DCIS, our study objects were relatively uniform and only focused on upgrading to IBC postoperatively. We assessed the clinical characteristics, radiologic features, and final pathological findings and identified that an IDC tumor size ≥ 0.5 cm on final pathology and the presence of LVI were independent risk predictors for SLN metastasis findings. To the best of our knowledge, this is the first study to explore the issues arising under the current general treatment guidelines. Moreover, the identified predictive factors in the current study may provide evidence regarding stratification in developing treatment strategies.

Many prior studies have attempted to identify the risk factors for SLN metastasis in patients with preoperative DCIS [7,9,12,14]. Several factors related to clinical characteristics, including young age at diagnosis, palpability, and multifocality, have been demonstrated to be associated with SLN metastasis, but none of them showed a consistent impact on positive SLN among those studies. In our series, unlike previous studies, none of the clinical features were related to SLN metastasis. A possible explanation is that the population composition of the previous studies included most DCIS and a relatively small number of underestimated IBC cases. In fact, the independent factors associated with SLN established by their researches and analyses are highly correlated with the presence of IBC, which has a major influence on SLN metastasis. Therefore, with the composition of our homogeneous study population with IBC, the above factors are relatively unable to achieve clinical significance. In our series, even if the patients who met the inclusion criteria of the COMET trial were classified as low-risk DCIS preoperatively, they still have an incidence of positive SLNs of 4% (2/50) [28], which was even slightly higher than the incidence of positive SLNs of 2.6% (5/192) for those who met the risk predictors of small IDC tumor size (≤0.5 cm) and absence of LVI.

In the present study, all factors related to the radiologic findings or biopsy procedures of breast tumors were also not related to SLN metastasis. Usually, different radiologic features represent distinctions in tissue components, histological type, and histological grade. In our series, according to the fact that the tumor size of DCIS in most lesions (312/316, 98.7%) was larger than that of IDC, it is reasonable to infer that its morphological presentation is still dominated by DCIS. Therefore, the previously identified risk factors associated with radiologic findings for underestimated IBC, such as lesion size and mass formation on mammography or ultrasonography may reflect the aggressiveness of DCIS and thus predict the existence of IBC, but it still lacks effectiveness in predicting SLN metastasis. In our opinion, the determinant factor for the occurrence of SLN metastasis should still be directly related to the nature of the tumor itself, rather than indirectly related to clinical or imaging manifestations.

Regarding the final pathology evaluation, all factors related to the features of DCIS cannot be used to predict the possibility of SLN metastasis. DCIS is a heterogeneous disease in terms of morphology, histology, and biology. In terms of DCIS architecture pattern, compared with non-comedo DCIS, comedo DCIS is recognized as more often associated with the presence of IBC and recurrence after treatment [29,30,31]. A similar situation can also be observed in tumor grades [22,32]. Although these factors have been recognized as being highly correlated with IBC, there is still no mandatory relationship among them. Once again, similar to clinical features or imaging manifestations, DCIS-related factors still cannot be predictors of SLN metastasis.

In the current study, only the pathologic findings of IDC included tumor size of IDC and LVI, which were confirmed as predictors of SLN metastasis. Moreover, the two identified factors were consistent in predicting SLN metastasis in asymptomatic or symptomatic patients. Many previous studies on underestimated IBC mentioned the positive correlation between the IBC found on final pathology and SLN metastasis, but only a few of them mentioned the impact of the size of IBC on SLN metastasis. Han et al. reported a retrospective analysis of 255 patients with preoperative DCIS and found that the size of IDC was significantly associated with positive SLN [7], which is consistent with our findings. In our study cohort, the T stage for most IDCs (91.8%) was T1. In many studies on predictors of lymph node metastasis for T1 tumors, the size of the tumor always had a determinant influence; however, whether T1a or T1b should be used as the boundary remains controversial [33,34,35]. In this study, an IDC tumor size of 0.5 cm as the cut-off point is indeed effective in predicting SLN metastasis; however, the optimal cut-off point for IDC tumor size may be revised after the inclusion of more cases.

Theoretically, the process of lymphatic system metastasis is considered to proceed in the order of lymphangiogenesis, then LVI, and finally lymph node metastasis [36]. LVI is a crucial step in breast cancer metastasis [37,38] and is only confirmed by pathological analysis. Numerous studies have previously demonstrated that LVI plays an important role in predicting ALN metastasis in patients with breast cancer [34,39,40,41,42]. Ozmen et al. observed that patients with LVI were more likely to have positive SLNs than those without LVI (51.3% vs. 30.3%, *p* = 0.004) [41]. Similarly, Gajdos et al. demonstrated a significant association between LVI and ALN metastasis in a retrospective study of 850 cases, in which positive lymph nodes were detected in 51% of the patients with LVI than in 19% of those without LVI [42]. In the present study, there was a statistically significant correlation between the presence of LVI and SLN metastasis, being present more frequently in SLN-positive patients than in SLN-negative patients (35.8% vs. 1%, *p* < 0.0001), which is consistent with the research findings mentioned above.

Several studies observed that IDC with accompanying DCIS had a trend to represent a favorable biology in comparison with pure IDC and showed that it was associated with low-grade tumors, smaller tumor size, and less lymph node involvement [43,44,45]. In a study of 3001 patients with pure IDC (79.4%) and IDC with accompanying DCIS (20.6%) reported by Goh et al., the incidence of LVI in IDC with accompanying DCIS was 7.6% [45]. Furthermore, the incidence of LVI as low as 2.7% (3/112) was documented in a study by Lyson et al. that reported 112 cases of DCIS with microinvasion [46]. These results imply that the smaller the IDCs, the less likely the presence of LVI. Considering that most of the IDCs in the current study were small tumors, 32% (101/316) of the overall cases were categorized as microinvasion (≤1 mm) and 76.9% (243/316) as T1b (≤1 cm); it was not surprising to observe such a low incidence of LVI (3.5%) in our series.

The most important role of SLNB in clinical lymph node-negative breast cancer is to provide information on treatment options, for example, whether a more extensive surgical procedure, such as ALN dissection, or more aggressive systemic drug therapy, such as chemotherapy or target therapy, is required. However, SLNB is never completely free of any side effects, such as bruising, pain, lymphedema, or impairment of upper extremity movement, which are all known possible side effects. Therefore, prospective studies are underway to confirm that certain low-risk groups will have a non-inferior prognosis without receiving SLNB [17,18,19]. In the current study, the final pathological evaluation of the resected breast specimen showed that, as long as the IDC tumor was > 0.5 cm or had a presence of LVI, the incidence of SLN metastasis would be so high that the omission of SLN was not allowed. In contrast, when no established risk predictor was presented on the final pathology in our series, breast cancer combined with SLN metastasis was as low as 2.6% (5/192), which made us cautiously consider the possibility of omitting SLNB. It is worth nothing that, if no sentinel lymph node biopsy would have been performed for patients without identified risk factors in our series, the administration of systemic therapy would be changed in five patients. The molecular types of the five missed patients were three luminal types, one triple negative type, and the remaining one HER2 positive type. Not surprisingly, the missing information on SLN metastasis would result in no adjuvant chemotherapy for triple negative breast cancer and neither adjuvant chemotherapy nor target therapy for HER2 positive breast cancer, which would be expected to have a greater impact on prognosis than luminal breast cancer with adjuvant hormone therapy. Under such circumstances, for those patients who had not received any adjuvant therapy, adopting more active and short-term follow-up examinations, including axillary lymph nodes as an alternative management, might be able to detect residual disease early, thereby reducing the adverse effects associated with missed diagnosis of nodal metastases.

Since the detection of IDC tumor size and LVI was highly dependent on the pathology of the resected specimen, it was still necessary to perform SLNB at the same time for cases expected to undergo total mastectomy. On the other hand, after BCS, in patients diagnosed with DCIS preoperatively by CNB, with no risk predictors in the final pathological evaluation, omitting SLNB may be an acceptable opinion. This view can be observed in our series of patients undergoing BCS in the absence of any predictor; their SLN metastasis rate was extremely low at 1.3%.

The current study had several limitations. First, since it was a retrospective analysis performed at a single institution, selection bias may have been inevitable. Despite the fact that our research was based on a reliable dataset derived from a relatively homogeneous and large number of cases, the generalization of the findings in our study may not be appropriate for all institutions. Further multi-institutional prospective studies are required. Second, it was difficult to accurately confirm how many cases of patients undergoing mastectomy could actually be replaced by BCS in the current study. In fact, among the patients who underwent mastectomy without any predictive factors in the present study, the four patients with SLN metastasis were accompanied by large DCIS, which made it impossible to perform BCS. This allowed us to reasonably infer that if the objective conditions of the tumor were not added to the patient’s preference for surgical procedure selection, more BCS cases without SLN metastasis would be added to the calculation and analysis, resulting in a lower incidence of SLN metastasis than the present value. Third, the overall median follow-up time was not long enough, so we could not assess whether the identified predictor of SLN metastasis in this study is also related to the prognosis of breast cancer.

## 5. Conclusions

Our study revealed that there were no reliable clinical or imaging features that could be used as a predictor of SLN metastasis in underestimated IDC patients with an initial diagnosis of DCIS by imaged-guided CNB. The current study demonstrated that only the features related to IDC on final pathological assessment, including an IDC tumor size > 0.5 cm and the presence of LVI, could be regarded as predictors of SLN metastasis. Considering the two identified risk factors, the overall incidence of SLN metastasis was very low in the absence of any predictor. Since the predictors established in this study could only be found on resected breast specimens, this study supported the current guidelines for SLNB at the time of mastectomy. For patients diagnosed with DCIS by CNB preoperatively, when invasive cancer is confirmed after BCS, we suggest that SLNB may be omitted for those without any predictors. However, further prospective studies are warranted. 

## Figures and Tables

**Table 1:** Clinicopathologic findings and radiologic features of the entire cohort.

Variable	No. of Cases	%
Age (years), median (IQR)	53 (16)	
Lesion location		
Left breast	180	57.0
Right breast	136	43.0
First-degree family history of breast cancer		
Yes	31	9.8
No	285	90.2
Palpability		
Palpable	174	55.1
Non-palpable	142	44.9
Lesion-detecting imaging modality		
Ultrasonography only	44	13.9
Mammography only	36	11.4
Detected by both ultrasonography and mammography	236	74.7
Radiologic morphology		
Mass with calcifications	164	51.9
Mass without calcifications	85	26.9
Non-mass	67	21.2
Parenchymal density		
Entirely fatty	11	3.5
Scattered fibroglandular	52	16.5
Heterogeneously dense	179	56.6
Extremely dense	74	23.4
MMG-BI-RADS category		
0	49	15.5
1	6	1.9
2	26	8.2
4a	50	15.8
4b	71	22.5
4c	78	24.7
5	36	11.4
Ultrasound-BI-RADS category		
1	15	4.7
2	10	3.2
3	10	3.2
4a	82	25.9
4b	72	22.8
4c	75	23.7
5	52	16.5
Image-guided procedure		
Ultrasound-guided	275	87.0
MMG-guided	41	13.0
Needle gauge		
7/10	38	12.0
14/16/18	278	88.0
Operation type		
Mastectomy	191	60.4
BCS	125	39.6
DCIS tumor size (cm), median (IQR)	3.3 (2.6)	
Tumor necrosis area		
None	36	11.4
Focal	178	56.3
Large	102	32.3
DCIS tumor grading		
Low	34	10.7
Intermediate	144	45.6
High	138	43.7
DCIS architecture pattern		
Comedo	122	38.6
Non-comedo	194	61.4
Lymphovascular invasion		
Present	11	3.5
Absent	305	96.5
IDC area		
Multifocal/multicentric	142	44.9
Unifocal	174	55.1
IDC ^a^		
T1mi	101	32.0
T1a	92	29.1
T1b	50	15.8
T1c	47	14.9
T2	26	8.2
IDC tumor grading		
1	81	25.6
2	133	42.1
3	44	13.9
Unknown	58	18.4
Estrogen receptor status		
Positive	197	62.3
Negative	119	37.7
Progesterone receptor status		
Positive	176	55.7
Negative	140	44.3
HER2 status		
Positive	110	34.8
Negative	206	65.2
Ki-67		
<20	221	69.9
≥20	70	22.2
Unknown	25	7.9

IQR, interquartile range; MMG, mammography; BI-RADS, Breast Imaging Reporting and Data System; BCS, breast-conserving surgery; DCIS, ductal carcinoma in situ; IDC, invasive ductal carcinoma. Figures are numbers with percentages in parentheses, unless otherwise stated. ^a^ T stage was defined according to the eighth edition of the American Joint Commission on Cancer TNM staging system for breast cancer.

**Table 2:** Clinicopathological findings and radiologic features of patients with sentinel lymph node metastasis.

Age (yrs)	Radiologic Morphology	Surgery Type	Final Pathology							
			LVI	DCIS (mm)	IDC (mm)	Molecular Subtype	No. of SLNs ^a^	pN by SLNB ^b^	No. of LN by ALND ^c^	Final TNM Stage ^b^
50	non-mass	Mast.	present	26	9	HER2 positive	1/1	pN1	1/17	pT1bN1aM0
38	mass without calcifications	Mast.	absent	37	9	Luminal A	1/1	pN1mi	0/39	pT1bN1miM0
54	mass without calcifications	BCS	absent	11	7	Luminal A	1/1	pN1	0/20	pT1bN1M0
55	mass with calcifications	Mast.	absent	70	<1	HER2 positive	1/2	pN1	0/12	pT1miN1aM0
42	mass with calcifications	Mast.	present	56	12	Luminal B/HER2 positive	1/2	pN1mi	0/20	pT1cN1miM0
69	mass with calcifications	Mast.	present	10	35	Luminal A	1/2	pN1	0/10	pT2N1aM0
41	mass with calcifications	Mast.	present	42	32	Luminal A	1/1	pN1	1/16	pT2N1aM0
41	mass with calcifications	Mast.	absent	6	8	Triple negative	1/1	pN1mi	NP	pT1bN1miM0
68	mass without calcifications	Mast.	absent	52	<1	Luminal A	1/5	pN0(i+)	NP	pT1miN0(i+)M0
59	mass without calcifications	BCS	absent	47	2.5	Triple negative	1/1	pN1	0/20	pT1aN1aM0
53	mass without calcifications	Mast.	absent	46	12	HER2 positive	1/1	pN1	0/44	pT1cN1aM0
42	mass without calcifications	Mast.	absent	60	19	Luminal A	1/1	pN1	NP	pT1cN1aM0
65	mass without calcifications	Mast.	absent	48	20	Luminal A	2/2	pN1	0/14	pT1cN1aM0
64	mass without calcifications	Mast.	present	25	45	Luminal A	1/2	pN1	1/28	pT2N1aM0
47	non-mass	Mast.	present	109	12	HER2 positive	1/2	pN1	0/20	pT1cN1aM0
74	mass with calcifications	Mast.	present	38	<1	HER2 positive	1/2	pN1	1/18	pT1miN1aM0
57	mass with calcifications	Mast.	absent	50	17	Luminal B HER2 negative	1/2	pN1mi	NP	pT1cN1miM0
37	mass with calcifications	BCS	absent	18	18	Luminal B/HER2 negative	1/2	pN1mi	NP	pT1cN1miM0
39	mass with calcifications	Mast.	present	47	39	Luminal B/HER2 negative	3/3	pN1	3/12	pT2N2aM0
42	mass with calcifications	BCS	absent	40	31	Luminal B/HER2 negative	2/3	pN1	11/27	pT2N3aM0
67	mass without calcifications	Mast.	absent	45	5	Luminal A	1/2	pN1mi	0/11	pT1aN1miM0
44	non-mass	Mast.	absent	47	1.6	Luminal B/HER2 negative	1/2	pN1	0/17	pT1aN1aM0
55	non-mass	BCS	absent	34	32	Luminal A	1/1	pN1mi	NP	pT2N1miM0

LVI, lymphovascular invasion; DCIS, ductal carcinoma in situ; IDC, invasive ductal carcinoma; SLNs, sentinel lymph nodes; SLNB, sentinel lymph node biopsy; ALND, axillary lymph node dissection; Mast., Mastectomy; HER2, human epidermal growth factor receptor 2; BCS, Breast conserving surgery; NP, not performed. ^a^ Values in table are numbers of sentinel lymph nodes removed (positive/examined). ^b^ Tumor size and lymph node status were categorized according to the eight edition of the American Joint Commission on Cancer TNM staging system for breast cancer. ^c^ Values in table are numbers of additional lymph nodes removed by subsequent axillary lymph node dissection (positive/examined).

**Table 3:** Univariate analysis of factors influencing the lymph node status in underestimated invasive breast. Carcinoma patients with initial diagnosis of ductal carcinoma in situ by image-guided core needle biopsy.

Variable	Lymph Node		*p* Value
	Negative (*n* = 293)	Positive (*n* = 23)	
Age (years), median (IQR)	53.0 (16.0)	53.0 (22.0)	
Lesion location			0.965
Left breast	167 (57.0)	13 (56.5)	
Right breast	126 (43.0)	10 (43.5)	
1st degree family history of breast cancer			0.146
Yes	31 (10.6)	0	
No	262 (89.4)	23 (100.0)	
Palpability			0.059
Palpable	157 (53.6)	17 (73.9)	
Non-palpable	136 (46.4)	6 (26.1)	
Lesion-detecting imaging modality			0.652
Ultrasonography only	42 (14.3)	2 (8.7)	
Mammography only	34 (11.6)	2 (8.7)	
Detected by both ultrasonography and mammography	217 (74.1)	19 (82.6)	
Radiologic morphology			0.389
Mass with calcifications	154 (52.6)	10 (43.5)	
Mass without calcifications	76 (25.9)	9 (39.1)	
Non-mass with calcifications	63 (21.5)	4 (17.4)	
Parenchymal density			0.070
Entirely fatty	8 (2.7)	3 (13.0)	
Scattered fibroglandular	49 (16.7)	3 (13.0)	
Heterogeneously dense	166 (56.7)	13 (56.5)	
Extremely dense	70 (23.9)	4 (17.4)	
MMG-BI-RADS category			0.492
0	43 (14.7)	6 (26.1)	
1	5 (1.7)	1 (4.3)	
2	25 (8.5)	1 (4.3)	
4a	49 (16.7)	1 (4.3)	
4b	65 (22.2)	6 (26.1)	
4c	72 (24.6)	6 (26.1)	
5	34 (11.6)	2 (8.7)	
Ultrasound-BI-RADS category			0.080
1	14 (4.8)	1 (4.3)	
2	10 (3.4)	0	
3	10 (3.4)	0	
4a	80 (27.3)	2 (8.7)	
4b	62 (21.2)	10 (43.5)	
4c	67 (22.9)	8 (34.8)	
5	50 (17.1)	2 (8.7)	
Image-guided procedure			0.751
Ultrasound-guided	254 (86.7)	21 (91.3)	
MMG-guided	39 (13.3)	2 (8.7)	
Needle gauge			>0.999
7/10	36 (12.3)	2 (8.7)	
14/16/18	257 (87.7)	21 (91.3)	
Operation type			0.070
Mastectomy	173 (59.0)	18 (78.3)	
BCS	120 (41.0)	5 (21.7)	
DCIS tumor size (cm), median (IQR)	3.3 (2.6)	4.5 (2.4)	0.117
DCIS tumor grading			0.797
Low	32 (10.9)	2 (8.7)	
Intermediate	132 (45.1)	12 (52.2)	
High	129 (44.0)	9 (39.1)	
DCIS architecture pattern			0.084
Comedo	117 (39.9)	5 (21.7)	
Non-comedo	176 (60.1)	18 (78.3)	
Tumor necrosis area			0.912
None	33 (11.3)	3 (13.0)	
Focal	166 (56.7)	12 (52.2)	
Large	94 (32.1)	8 (34.8)	
IDC area			0.884
Multiple	132 (45.1)	10 (43.5)	
Single	161 (54.9)	13 (56.5)	
IDC tumor size (cm)			<0.001
≦0.5	187 (63.8)	6 (26.1)	
>0.5	106 (36.2)	17 (73.9)	
IDC tumor grading			0.165
1	78 (26.6)	3 (13.0)	
2	119 (40.6)	14 (60.9)	
3	40 (13.7)	4 (17.4)	
Unknown	56 (19.1)	2 (8.7)	
Lymphovascular invasion			<0.0001
Present	3 (1.0)	8 (34.8)	
Absent	290 (99.0)	15 (65.2)	
Estrogen receptor status			0.458
Positive	181 (61.8)	16 (69.6)	
Negative	112 (38.2)	7 (30.4)	
Progesterone receptor status			0.340
Positive	161 (54.9)	15 (65.2)	
Negative	132 (45.1)	8 (34.8)	
HER2 status			0.647
Positive	103 (35.2)	7 (30.4)	
Negative	190 (64.8)	16 (69.6)	
Ki-67			0.873
<20	206 (70.3)	15 (65.2)	
≧20	64 (21.8)	6 (26.1)	
Unknown	23 (7.8)	2 (8.7)	

IQR, interquartile range; MMG, mammography; BI-RADS, Breast Imaging Reporting and Data System; BCS, breast-conserving surgery; DCIS, ductal carcinoma in situ; IDC, invasive ductal carcinoma; HER2, human epidermal growth factor receptor 2. Figures are numbers with percentages in parentheses, unless otherwise stated.

**Table 4:** Multivariate analysis of factors influencing the lymph node status in underestimated invasive breast carcinoma patients with initial diagnosis of ductal carcinoma in situ by image-guided core needle biopsy.

Variable	Odds Ratio	95% Confidence Interval of Odds	*p* Value
IDC tumor size (cm)			
≤0.5	1		
>0.5	3.11	1.09–8.81	0.033
Lymphovascular invasion			
Present	32.85	7.56–142.80	<0.0001
Absent	1		

IDC, invasive ductal carcinoma.

## Data Availability

The data presented in this study are available on request from the corresponding author. The data are not publicly available due to ethical regulations.

## Supplementary Materials

The following are available online at https://www.mdpi.com/article/10.3390/cancers13164099/s1. Table S1: Univariate analysis of factors influencing the lymph node status in asymptomatic breast cancer patients.
