# Supplementary material for: Predictors of Sentinel Lymph Node Metastasis in Postoperatively Upgraded Invasive Breast Carcinoma Patients
Source: Cancers (Basel). 2021 Aug 14;13(16):4099. doi: 10.3390/cancers13164099 (PMC8392104; doi:10.3390/cancers13164099)
Supplement: Supplementary file 1 [file cancers-13-04099-s001.zip › cancers-1298192-supplementary.pdf]

Table S1. Univariate analysis of factors influencing the lymph node status in asymptomatic breast cancer patients.

Variable	Lymph node		*p* value
	Negative (*n* = 115)	Positive (*n* = 5)	
Age (years), median (IQR)	53 (12)	50 (12)	0.156
Lesion location			>0.999
Left breast	68 (59.1)	3 (60.0)	
Right breast	47 (40.9)	2 (40.0)	
1st degree family history of breast cancer			>0.999
Yes	12 (10.4)	0	
No	103 (89.6)	5 (100.0)	
Lesion-detecting imaging modality			>0.999
Mammography only	33 (28.7)	1 (20.0)	
Detected by both ultrasonography and mammography	82 (71.3)	4 (80.0)	
Radiologic morphology			0.279
Mass with calcifications	54 (47.0)	1 (20.0)	
Mass without calcifications	9 (7.8)	1 (20.0)	
Non-mass with calcifications	52 (45.2)	3 (60.0)	
Parenchymal density			>0.999
Entirely fatty	2 (1.7)	0	
Scattered fibroglandular	21 (18.3)	1 (20.0)	
Heterogeneously dense	69 (60.0)	3 (60.0)	
Extremely dense	23 (20.0)	1 (20.0)	
MMG-BI-RADS category			0.497
0	9 (7.8)	1 (20.0)	
4a	30 (26.1)	0	
4b	30 (26.1)	2 (40.0)	
4c	42 (36.5)	2 (40.0)	
5	4 (3.5)	0	
Ultrasound-BI-RADS category			>0.999
1	13 (11.3)	1 (20.0)	
2	10 (8.7)	0	
3	10 (8.7)	0	
4a	34 (29.6)	0	
4b	26 (22.6)	1 (20.0)	
4c	18 (15.7)	1 (20.0)	
5	4 (3.4)	2 (40.0)	

Image-guided procedure			>0.999
Ultrasound-guided	77 (67.0)	3 (60.0)	
MMG-guided	38 (33.0)	2 (40.0)	
Needle gauge			0.643
7/10	35 (30.4)	2 (40.0)	
14/16/18	80 (69.6)	3 (60.0)	
Operation type			0.649
Mastectomy	48 (41.7)	3 (60.0)	
BCS	67 (58.3)	2 (40.0)	
DCIS tumor size (cm), median (IQR)	2.8 (1.8)	2.6 (3.2)	0.486
DCIS tumor grading			0.315
Low	13 (11.3)	1 (20.0)	
Intermediate	57 (49.6)	1 (20.0)	
High	45 (39.1)	3 (60.0)	
DCIS architecture pattern			>0.999
Comedo	51 (44.3)	2 (40.0)	
Non-comedo	64 (55.7)	3 (60.0)	
Tumor necrosis area			0.086
None	4 (3.5)	1 (20.0)	
Focal	81 (70.4)	2 (40.0)	
Large	30 (26.1)	2 (40.0)	
IDC area			0.884
Multiple	46 (40.0)	2 (40.0)	
Single	69 (60.0)	3 (60.0)	
IDC tumor size (cm)			0.038
≤0.5	80 (69.6)	1 (20.0)	
>0.5	35 (30.4)	4 (80.0)	
IDC tumor grading			0.669
1	31 (27.0)	2 (40.0)	
2	49 (42.6)	2 (40.0)	
3	14 (12.1)	1 (20.0)	
Unknown	21 (18.3)	0	
Lymphovascular invasion			0.042
Present	0	1 (20.0)	
Absent	115 (100.0)	4 (80.0)	
Estrogen receptor status			>0.999
Positive	77 (67.0)	3 (60.0)	

Negative	38 (33.0)	2 (40.0)	
Progesterone receptor status			>0.999
Positive	68 (59.1)	3 (60.0)	
Negative	47 (40.9)	2 (40.0)	
HER2 status			>0.999
Positive	39 (33.9)	2 (40.0)	
Negative	76 (66.1)	3 (60.0)	
Ki-67			0.383
<20	82 (71.3)	3 (60.0)	
≥20	25 (21.7)	1 (20.0)	
Unknown	8 (7.0)	1 (20.0)	

IQR, interquartile range; MMG, mammography; BI-RADS, Breast Imaging Reporting and Data System; BCS, breast-conserving surgery; DCIS, ductal carcinoma in situ; IDC, invasive ductal carcinoma; HER2, human epidermal growth factor receptor 2. Figures are numbers with percentages in parentheses, unless otherwise stated.
